# Supplementary material for: Structural basis of siRNA recognition by TRBP double‐stranded RNA binding domains
Source: EMBO J. 2018 Feb 15;37(6):e97089. doi: 10.15252/embj.201797089 (PMC5852647; doi:10.15252/embj.201797089)
Supplement: Supplementary file 1 — Appendix [file EMBJ-37-e97089-s001.pdf]

Appendix Supplementary Information

Structural basis of siRNA recognition by TRBP double-stranded RNA binding domains

Authors

Gregoire Masliah[1], Christophe Maris[1], Sebastian L.B. König[2], Maxim Yulikov[3], Florian Aeschimann[4], Anna L. Malinowska[5], Julie Mabille[4], Jan Weiler[4], Andrea Holla[2], Juerg Hunziker[4], Nicole Meisner-Kober[4], Benjamin Schuler[2], Gunnar Jeschke[3], Frederic H.-T. Allain[1]

Affiliations

[1]Institute of Molecular Biology and Biophysics, ETH Zürich, CH-8093 Zürich, Switzerland
[2]University of Zürich, Department of Biochemistry, Winterthurerstrasse 190, CH-8057 Zürich, Switzerland
[3]Laboratory of Physical Chemistry, ETH Zürich, CH-8093 Zürich, Switzerland
[4]Novartis Institutes for Biomedical Research, CH-4000 Basel, Switzerland
[5]Institute of Pharmaceutical Sciences, Department of Chemistry and Applied Biosciences, ETH Zürich, CH-8093 Zürich, Switzerland.

Correspondence: allain@mol.biol.ethz.ch

Page	Description
A2	Appendix Figure S1
A5	Appendix Table S1
A6	Appendix Table S2
A7	Appendix Table S3
A8	Appendix Methods
A16	Appendix References

A

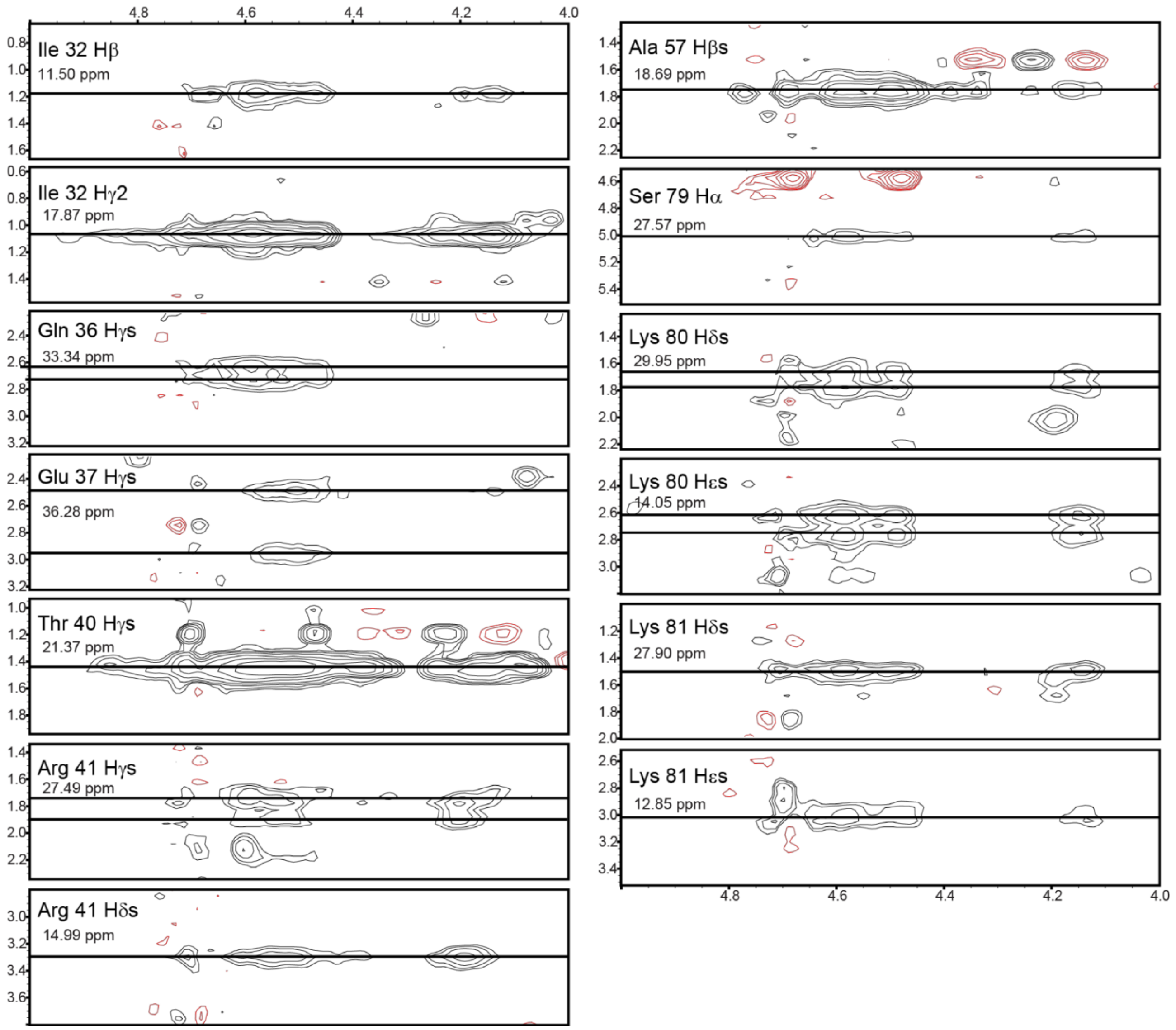

Ile 32 Hβ
11.50 ppm
Ile 32 Hγ2
17.87 ppm
Gln 36 Hγs
33.34 ppm
Glu 37 Hγs
36.28 ppm
Thr 40 Hγs
21.37 ppm
Arg 41 Hγs
27.49 ppm
Arg 41 Hδs
14.99 ppm
Ala 57 Hβs
18.69 ppm
Ser 79 Hα
27.57 ppm
Lys 80 Hδs
29.95 ppm
Lys 80 Hεs
14.05 ppm
Lys 81 Hδs
27.90 ppm
Lys 81 Hεs
12.85 ppm
4.8
4.6
4.4
4.2
4.0

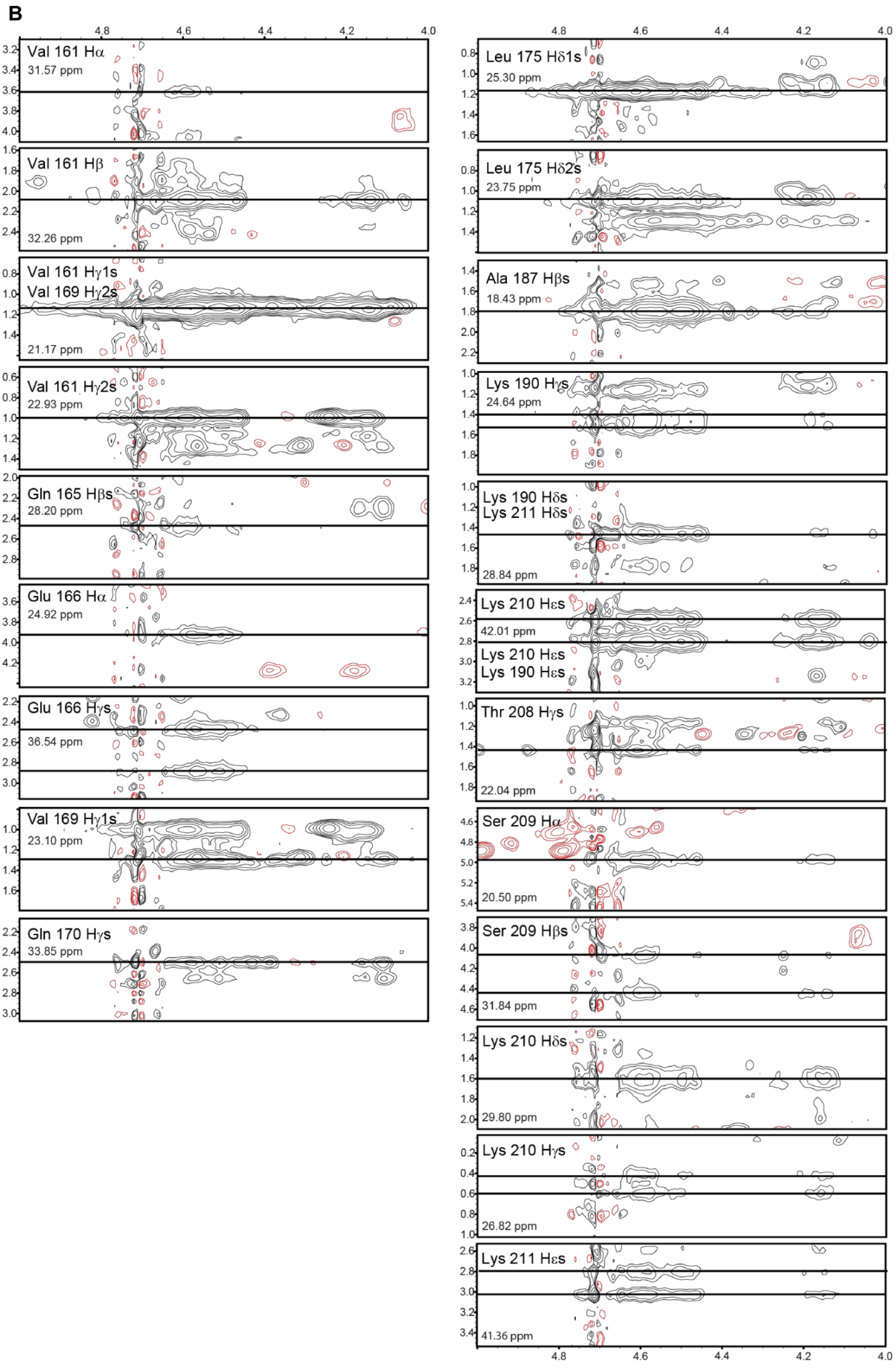
B
Val 161 Hα
31.57 ppm
Val 161 Hβ
32.26 ppm
Val 161 Hγ1s
Val 169 Hγ2s
21.17 ppm
Val 161 Hγ2s
22.93 ppm
Gln 165 Hβs
28.20 ppm
Glu 166 Hα
24.92 ppm
Glu 166 Hγs
36.54 ppm
Val 169 Hγ1s
23.10 ppm
Gln 170 Hγs
33.85 ppm
Leu 175 Hδ1s
25.30 ppm
Leu 175 Hδ2s
23.75 ppm
Ala 187 Hβs
18.43 ppm
Lys 190 Hγs
24.64 ppm
Lys 190 Hδs
Lys 211 Hδs
28.84 ppm
Lys 210 Hεs
42.01 ppm
Lys 210 Hεs
Lys 190 Hεs
Thr 208 Hγs
22.04 ppm
Ser 209 Hα
20.50 ppm
Ser 209 Hβs
31.84 ppm
Lys 210 Hδs
29.80 ppm
Lys 210 Hγs
26.82 ppm
Lys 211 Hεs
41.36 ppm

C

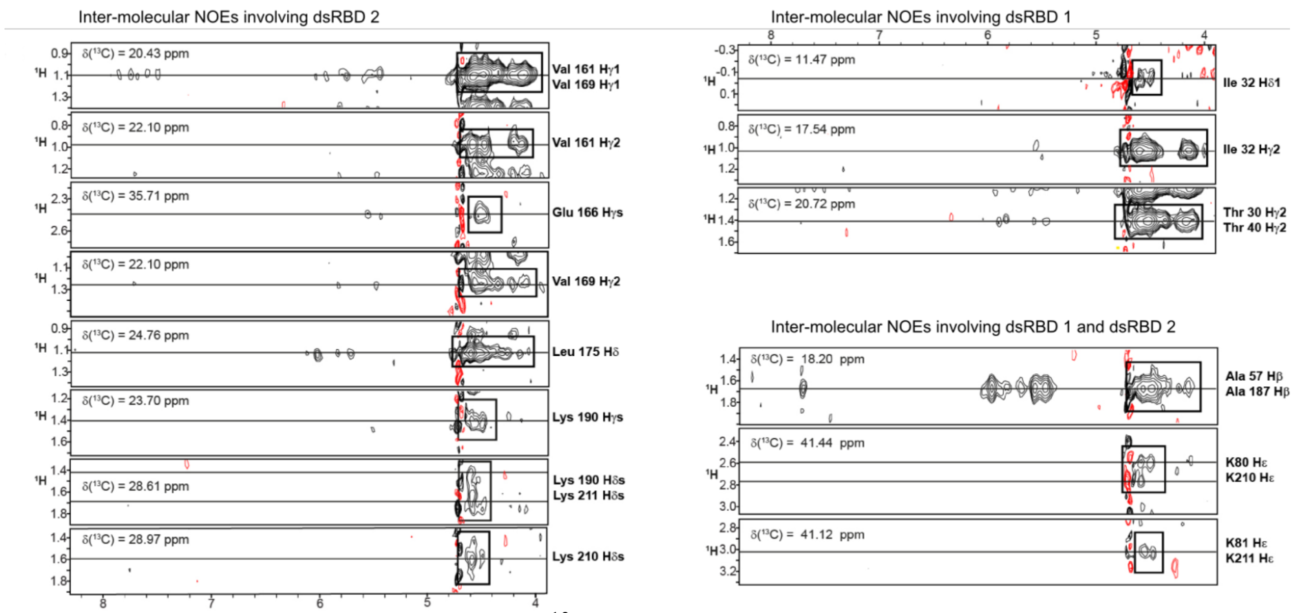

Appendix Figure S1. Selected regions of ^{13}C-edited-filtered NOESY spectra showing protein-RNA intermolecular NOEs observed in **(A)** the dsRBD1-EL86 complex, **(B)** the dsRBD2-EL86 complex, and **(C)** the dsRBD12-EL86 complex. The protein residue and proton involved in each intermolecular NOE are indicated on the right side the spectrum strips. The spectral window displayed in (A) and (B) corresponds to RNA H4'/H5'/H5", and to H6/H8/H2/H1'/H4'/H5'/H5" in (C).

NMR statistics of the structures of individual TRBP dsRBDs in dsRNA bound state [a,b]		
	**dsRBD1**	**dsRBD2**
NMR restraints		
Total NOE	945	1355
Intra-residue	200	318
Sequential (| i - j | = 1)	114	340
Medium range (1 < | i - j | < 5)	198	287
Long-range (| i - j | > 5)	433	410
Torsion angles[c]	126	96
Residual Dipolar Couplings[d]	44	217
Structure statistics		
Violations (mean ± SD)		
Distance restraints violations > 0.3 Å	1.2 ± 0.7	0.5 ± 0.5
Max. distance restraints violation (Å)	0.34 ± 0.05	0.31 ± 0.03
Dihedral angle violation > 5°	0.7 ± 0.6	0.0 ± 0.0
Max. dihedral angle violation (°)	6.3 ± 2.1	1.6 ± 0.9
RDC r.m.s.d.	1.0 ± 0.1	2.4 ± 0.1
Q-factor	0.05 ± 0.04	0.22 ± 0.00
Deviations from idealized geometry		
Bond lengths (Å)	0.0037 ± 0.0000	0.0038 ± 0.0000
Bond angles (°)	1.698 ± 0.013	1.538 ± 0.013
R.m.s.d. from averaged structure[e] (Å)		
Backbone	0.35 ± 0.11	0.22 ± 0.05
Heavy atoms	0.74 ± 0.08	0.81 ± 0.08
Ramachandran analysis		
Most favored regions (%)	93.5 ± 1.3	94.6 ± 1.2
Additionally allowed regions (%)	6.2 ± 1.0	5.4 ± 1.2
Generously allowed regions (%)	0.1 ± 0.3	0.0 ± 0.0
Disallowed regions (%)	0.2 ± 0.5	0.0 ± 0.0

Appendix Table S1.

a. NMR spectra were acquired on samples with a protein-RNA stoichiometry of 1:2.

b. All statistics were calculated on an ensemble of 20 structures of lowest energy.

c. Dihedral angle restraints were obtained with the program TALOS+ + [Shen et al. 2009].

d. dsRBD1 dataset consisted of 36 (NH) RDCs only. DsRBD2 dataset consisted of 55 (NH), 59 (CαC), 51 (HC), and 52 (NC) RDCs.

e. Structures from dsRBD1 and dsRBD2 ensembles were superposed, using residues 18-97 and 160-226, respectively.

Statistics on RDCs collected in the dsRBD12 - EL86 complex				
	Rp [a]	**r.m.s.d.**	**Da (Hz)**	**Rh**
**Individual domains** [b]				
**rbd 1**	0.93±0.02	4.24±0.67	10.33±0.48	0.25±0.05
**rbd 2**	0.95±0.02	3.41±0.58	10.84±0.44	0.29±0.05
**Two domains antiparallel** [c]				
**12,13** [d]	0.71±0.02	8.0±0.2	-1.15±6.04	0.61±0.04
**13,14** [d]	0.83±0.02	6.2±0.4	7.99±0.4	0.39±0.05
**14,15** [d]	0.93±0.02	4.1±0.5	10.44±0.19	0.25±0.03
**15,16** [d]	0.84±0.03	6.2±0.6	9.33±0.44	0.31±0.06
**Two domains parallel** [c]				
**37,38** [d]	0.93±0.01	4.1±0.4	10.35±0.31	0.27±0.04
**38,39** [d]	0.87±0.02	5.6±0.4	8.26±0.36	0.29±0.04
**39,40** [d]	0.71±0.03	8.0±0.3	1.34±5.71	0.59±0.05

Appendix Table S2.

a. Pearson's correlation factor between experimental and back-calculated RDCs

b. All values were calculated using the NMR structures of the single domains in their RNA bound form.

c. All values were calculated using three-dimensional models of dsRBD12-EL86 complexes obtained by keeping dsRBD2 at a fixed position while shifting dsRBD1 position systematically.

d. Sequence number of the RNA residue flanking Ala 57 methyl group (dsRBD1-loop 2).

Appendix Table S3. NMR statistics of the dsRBD12 - EL86 structure ensembles A and B

Structures of dsRBD12 – EL86 complex [a,b]			
NMR restraints	**dsRBD1**	**dsRBD2**	**EL86**
Total NOE	945	1355	268
Intra-residue	200	318	168
Sequential (| i - j | = 1)	114	340	120
Medium range (1 < | i - j | < 5)	198	287	0
Long-range (| i - j | > 5)	433	410	0
Hydrogen bonds	0	0	70
Intermolecular NOE	14	30	
Torsion angles[c]	126	96	240
Residual Dipolar Couplings[d]	44	217	0

Structures statistics	**Ensemble A**	**Ensemble B**
Violations (mean ± SD)		
Distance restraints violations > 0.4 Å	1.2 ± 0.8	0.5 ± 0.7
Max. distance restraint violation (Å)	0.50 ± 0.11	0.39 ± 0.05
Dihedral angle violation > 5°	4.8 ± 1.6	4.6 ± 1.0
Max. dihedral angle violation (°)	12.3 ± 9.3	9.1 ± 2.1
RDC r.m.s.d.	0.9 ± 0.1 / 3.1 ± 0.2	0.9 ± 0.1 / 3.1 ± 0.1
Q-factor	0.04 ± 0.01 / 0.29 ± 0.02	0.04 ± 0.01 / 0.29 ± 0.01
Deviations from idealized geometry		
Bond lengths (Å)	0.0037 ± 0.0000	0.0037 ± 0.0000
Bond angles (°)	1.845 ± 0.009	1.847 ± 0.007
Structural r.m.s.d.[e] (Å)		
Backbone	0.85 ± 0.13	0.88 ± 0.20
Heavy atoms	1.19 ± 0.11	1.25 ± 0.16
Ramachandran analysis		
Most favored regions (%)	91.8 ± 1.1	93.0 ± 1.0
Additionally allowed regions (%)	7.1 ± 0.9	5.9 ± 0.8
Generously allowed regions (%)	0.7 ± 0.6	0.5 ± 0.6
Disallowed regions (%)	0.4 ± 0.6	0.6 ± 0.6

a. NMR spectra were acquired on samples with a protein-RNA stoichiometry of 1:2.

b. All statistics were calculated on an ensemble of 20 structures of lowest energy.

c. Dihedral angle restraints for dsRBD1 and dsRBD2 were obtained with the program TALOS+ [Shen et al. 2009]. Standard values for a A-form RNA double helix were used for EL86.

d. DsRBD1 dataset consisted of 36 (NH) RDCs only. DsRBD2 dataset consisted of 55 (NH), 59 (CαC), 51 (HC), and 52 (NC) RDCs.

e. Structures from ensembles A and B were superposed, using residues 18-97 (dsRBD1), 160-225

(dsRBD2), and 1-19, 22-40 (EL86). Structures in complexes A and B were superposed on structures 10 and 17, respectively

Appendix Supplementary Methods

Structure calculation

Protein backbone and side chain resonance assignments were obtained from triple resonance spectra (HNCACB, CBCA(CO)NH, and HNCO) and TOCSY spectra (H(CCO)NH and (H)C(CO)NH), respectively. Inter proton distance restraints were extracted for each domain (dsRBD1 and dsRBD2) from 3D ^{13}C-edited NOESY-HSQC and 3D ^{15}N-edited NOESY-HSQC measured on dsRBD1-EL86 and dsRBD2-EL86 complexes (120 ms mixing times). Residual Dipolar Couplings (RDCs) were derived from the difference of peak splitting observed under isotropic and anisotropic (10 mg.mL^{-1} Pf1 phages (ASLA biotech) conditions. The set of RDCs used to orient dsRBD1 and dsRBD2 with respect to each other was measured on the dsRBD12-EL86 complex. Aromatic-aromatic, sugar-aromatic and imino-imino NOE cross peaks observed in NOESY spectra recorded on EL86 were typical of those found in A-form RNA structures [Buuren et al., 1998; Wüthrich 1986] The two uracils at both 3'overhangs were flexible as indicated by the intense H5-H6 correlations in TOCSY spectrum (data not shown). The NOE pattern typical of an A-form RNA and the flexibility of the nucleotides at both 3' overhangs remained unchanged upon protein binding. Based on these data we used both inter proton distance and dihedral restraints to constraint EL86 to an A-form RNA helix. Protein chemical shifts and NOESY spectra were used as input to the programs ATNOS-CANDID [Herrmann et al., 2002] and CYANA [Güntert 2004]. Structures were further refined with the AMBER 12 package [Case et al.] using a standard simulated-annealing protocol. *Residual Dipolar Coupling (RDC) analysis.* The fourteen structural models of the dsRBD12-EL86 complex used to analyze RDCs, were calculated with the program CYANA and a combination of experimental and artificial structural constraints. The two dsRBDs were folded using experimental distance restraints and RDCs collected on the single domains in complex with EL86, EL86 was folded using inter proton and backbone dihedral restraints with standard values for a A-form RNA double helix (Supplementary Methods), dsRBD1 and dsRBD2 were positioned on EL86 using inter proton distance restraints. The RNA residues and the protein

side chains involved in these restraints were selected on the basis of inter molecular NOEs. The set of RNA residues serving as docking points for each dsRBD was changed consistently for each model in order to preserve dsRBD's canonical dsRNA binding mode. The agreement of each model with a set of RDCs measured on the dsRBD12-EL86 complex was estimated by calculating the r.m.s.d. and the Pearson correlation factor

For each model, the r.m.s.d. and the Pearson correlation factor between calculated and experimental RDCs were calculated with the program PALES [Zweckstetter 2008].

Protein purification

E.coli BL21(DE3) Codon-plus (RIL) cells were transformed with pet28a vectors allowing the expression of proteins fused to a polyhistidine affinity tag at the N-terminus. The pet28a plasmids were engineered to encode a TEV cleavage site between the polyhistidine affinity tag and the multicloning site. Cells were grown in LB medium or M9 medium supplemented with ^{13}C-labeled glucose and / or $^{15}NH_4Cl$ (for isotopically labeled samples) at 37 °C to a cell density of 0.6 a.u. (OD_{600}). Protein expression was induced by addition of 0.5 mM of isopropyl-β-D-thiogalactopyranoside, temperature was cooled down to 30 °C, cells were harvested after 12-16 hours and resuspended in buffer A (50 mM Tris-HCl [pH 8.0], 1 M NaCl, 7 mM β-mercaptoethanol, 10 mM imidazole, 0.1% Triton 100X) and lysed by sonication. The lysate was clarified by centrifugation (30,000 × g, 40 min at 4 °C), the supernatant was loaded on a column prepacked with Ni-NTA beads (GE Healthcare) previously equilibrated with buffer A. The proteins were eluted with a linear gradient of buffer B (50 mM Tris-HCl [pH 8.0], 1 M NaCl, 7 mM β-mercaptoethanol, 1 M imidazole). The fractions containing the protein were pooled and dialyzed overnight at 4 °C against buffer C (20 mM $NaPO_4$ [pH 6.4], 7 mM β-mercaptoethanol). Precipitate was discarded by centrifugation. Polyhistidine tags were cleaved off with in-house TEV protease (1 OD_{280} per 100 OD_{280} of substrate, overnight at 4°C), uncleaved products were removed by further processing the samples through Ni-NTA beads. As a final purification step, samples were applied on a Superdex

200pg 16×60 column (GE Healthcare) equilibrated with NMR buffer (20 mM $NaPO_4$ [pH 6.4], 5 mM DTT). Protein-containing fractions were pooled and concentrated to 1.5 mM and stored at -20 ℃ until further use.

For single-molecule FRET experiments, the codon-optimized sequence of TRBP 22-235 with amino acid exchanges C73A/M100C/C196V and a threonine insertion between C158 and N159 was cloned into a pET47b(+)-based vector. Site-directed mutagenesis was performed to create single-cysteine variants at position 100 (dsRBD12 M100C/C158S) and position 158 (dsRBD12 M100S). For fluorescence labeling, maleimide-functionalized CF660R (Biotium, Inc., Fremont, CA) dissolved in anhydrous DMSO was added in 3-fold excess to purified protein solution. The coupling reaction was allowed to proceed overnight at 8°C, followed by quenching with 10 mM DTT. Free dye and DTT were removed using a HiTrap Desalting column (GE Healthcare Bio-Sciences AG, Uppsala, Sweden). Finally, singly-labeled dsRBD12 variants were separated from unlabeled species by cation-exchange chromatography (MonoS 5/50 GL, GE Healthcare Bio-Sciences AG, Uppsala, Sweden). To generate donor-acceptor labeled dsRBD12, maleimide-functionalized CF660R was added at a molar ratio of 1:0.7 (protein:dye). The reaction was performed and quenched as described above, followed by separation of singly labeled TRBP from unlabeled and doubly labeled protein by cation-exchange chromatography. Subsequently, maleimide-functionalized Cy3b was added at a molar ratio of 1:3 (protein:dye). The coupling reaction was conducted as described above and doubly labeled TRBP purified by cation-exchange chromatography. For all proteins, sample quality and chromophore incorporation were confirmed by electrospray ionization mass spectrometry.

EPR Spectroscopy

Nitroxide–nitroxide distance measurements were performed with the four-pulse DEER experiment [Pannier et al., 2000] at Q band (34–36 GHz) at 50 K on home-built EPR spectrometer [Gromov et al., 2001] with a rectangular cavity allowing oversized samples [Tschaggelar et al.

2009]. The chosen DEER measurements temperature of 50 K corresponds approximately to the optimum measurement conditions with respect to the longitudinal and transverse relaxation of nitroxide radicals. The sample temperature was stabilized with a He-flow cryostat (ER 4118 CF, Oxford Instruments).

The previously reported optimal settings were used in the DEER measurements [Polyhach et al., 2012]. In brief, all pulses were set to a duration of 12 ns. The offset between pump and detection frequencies was set to $f_{det} - f_{pump} = -100\text{MHz}$. The first inter-pulse delay time in the DEER sequence was set to 400 ns in all cases. The second delay time (between the primary echo and the refocusing pulse) was set according to the length of the DEER trace required to provide sufficient range for background correction. Typical measurement time was 5–20 h depending on the length of the DEER trace, and actual dipolar modulation depth.

Fitting of DEER data was performed with DeerAnalysis 2009 software [Jeschke et al. 2006]. To avoid possible artefacts, the range for the background fit was cut at least 100 ns before the end of the DEER trace. All traces were fitted with the unrestricted distance distribution model. In each case Tikhonov regularization [Jeschke et al., 2004; Chiang et al., 2005] was performed and distributions corresponding to different regularization parameters were analyzed. A 3D background model was assumed in the background fits for all samples. In all cases, the obtained distance distribution was stable with respect to the change of the background model.

RNA synthesis and labeling

Single-molecule FRET experiments: EL86 oligonucleotides were purchased RP-HPLC-purified from Integrated DNA Technologies BVBA (Leuven, Belgium), where EL86up (5'-UUA AUU AUC UAU UCC GUA CUU-3') was functionalized with a biotin at its 3'-end, while a primary amino modifier was incorporated at the 3'-end of EL86down (5'-GUA CGG AAU AGA UAA UUA AUU-3'). For both sequences, the purity was confirmed by analytical RP-HPLC, followed by fluorescently labeling EL86down with *N*-hydroxysuccinamide-functionalized Cy3B (GE

Healthcare, Glattbrugg, Switzerland) [Greenfeld & Herschlag 2013]. Cy3B-labelled EL86down was EtOH-precipitated to remove free dye, followed by preparative RP-HPLC purification and lyophilization. Successful incorporation of Cy3B was confirmed by MALDI-MS. All RNA sequences were dissolved in nuclease-free H_2O (Applichem GmbH, Darmstadt, Germany) and stored at -20 °C at a concentration of 100 μM until further use. *NMR and EPR experiments:* EL86 antisense (5'-UUA AUU AUC UAU UCC GUA CUU-3') and sense (5'-GUA CGG AAU AGA UAA UUA AUU-3') strands were synthetized on an Äkta Oligopilot plus OP100 (*GE Healthcare*) with a 12 mL column reactor (*GE Healthcare*, order no. 18-1101-16) using a solid-phase synthesis cyclic procedure consisting of 5 steps, based on the manufacturer's template method. Base-protected ribonucleoside phosphoramidites were dissolved in anhydrous acetonitrile under argon to a concentration of 0.15 M. Activated molecular sieves was added to each amidite solution and the bottles were left to stand overnight. The following synthesis cycle applied: First, the 4,4'-dimethoxytrityl (DMT) protecting group was removed by adding deblocking reagent (3% DCA in toluene, *Merck*, order no. BI0832). Coupling was then achieved by adding the phophoramidite solution (1.9 eq.) to the column along with an equal volume of 0.5M 5-ethylthiotetrazole (*ChemGenes*, order no. RN-6397) in acetonitrile. Coupling was followed by capping by adding Cap A (*Biosolve*, order no. 036124, 2-methylimidazole / acetonitrile 1:4 (v/v)) and Cap B, obtained by mixing equivalent amounts of Cap B1 (*Biosolve*, order no. 037424, acetic anhydride / acetonitrile 2:3 (v/v)) and Cap B2 (*Biosolve*, order no. 037425, pyridine / acetonitrile 3:2 (v/v)). The oligonucleotide was subsequently oxidized by adding iodine (*Biosolve*, order no. 150724, 0.05M iodine in pyridine/water 9:1 (v/v)). The column was washed between each step with anhydrous acetonitrile. The synthesis procedure ended with a final detritylation step in order to remove the last DMT-group, allowing for easier purification. After completion of synthesis, the column was placed under vacuum in order to remove all remaining acetonitrile, and the support was transferred into a glass vial.

The oligonucleotide was cleaved from the support and deprocted by first incubating the support in a

glass bottle with 19 mL concentrated aqueous methylamine at 35°C for 45 min under gentle agitation. Then the flask was cooled in an ice bath and the solid support filtered off over a sintered glass funnel. The support was washed with DMSO (3 x 10 mL). The filtrate was cooled in an ice bath and 15 mL triethylamine-trishydrofluoride was added slowly under shaking. The solution was heated to 50°C for 60 min and then cooled again in an ice bath. When cold, 64 mL of a 50 mM sodium acetate buffer (pH=5.2) was added.

The oligonucleotide was purified on a Fineline pilot 35 column (*GE Healthcare*, order no. 18-1102-02) filled with TSK Gel SuperQ-5PW ion exchange resin (*Tosoh*, order no. 18546) using an ÄKTAexplorer (*GE Healthcare*). Buffer A: 20 mM aqueous sodium phosphate, pH = 7.0; buffer B: 20 mM aqueous sodium phosphate, pH = 7.0, 1M NaCl; gradient: 20 – 80% B in 43 column volumes; flow rate 30 mL / min; column heated to 63°C. Fractions of appropriate purity were pooled and then desalted by tangential flow filtration (TFF) on a Minim II TFF system (*Pall Corporation*) using a 1K Omega Centramate T-Series filtration unit (*Pall Corporation*, order no. OS001T12). The oligonucleotide was filtered on a Steriflip-GP filtration unit (*Millipore*, order no. SCGP00525) and the quality of the oligonucleotide verified by HPLC and UPLC-MS before lyophilization.

Analytical ion-exchange HPLC was performed on an *Agilent* 1200 Series HPLC with a Dionex DNAPac® PA200 analytical column (4 x 250 mm). Buffer A: 10 mM $NaClO_4$, 10 mM Tris-HCl, 20% EtOH; buffer B: 300 mM $NaClO_4$, 10 mM Tris-HCl, 20% EtOH; flow rate: 1mL/min; column temperature: 80°C; gradient: 40 – 60% B in 7 min.

UPLC-MS chromatography analysis was performed on an Acquity UPLC / LCT Premier XE ES mass spectrometer (*Waters*) with an Acquity UPLC® BEH C18 1.7 µm 2.1 x 50 mm column. Buffer A: triethylamine (32 mM); hexafluoroisopropanol (100 mM) in water; buffer B: 20% buffer A, 80% MeOH; flow rate: 0.25 mL/min; column temperature: 15°C; gradient: 5 – 60% B in 3.75 min.

mRNA knockdown assay

Transfection of Hela cells with siRNAs. The human HeLa YFP cells were maintained in 5 % humidified CO2 atmosphere at 37 °C in antibiotics free DMEM medium (Invitrogen, #21063) supplemented with + 10% FCS (heat-inactivated) and + 1% L-Glu designated as growth medium. After reaching an 80 % subconfluent stage, one day before transfection, cells were harvested by trypsinization and finally seeded in 150 µl DMEM growth medium at a density of 4000 cells/well into black 96-well assay plates (Corning Costar, clear bottom, #3606) and incubated in a 5 % humidified CO2 atmosphere at 37 °C. After overnight incubation, the cells were transfected with siRNA-HiPerFect complex. Briefly, HiPerFect and siRNA were pre-diluted in OptiMEM. The siRNA-HiPerFect mixture (18.3 nM siRNA, 0.450 µl HiPerFect in 65.45 µl) were pre-incubated for 18 minutes to form the siRNA-HiPerFect complex. Just before adding the complex, 50 µl of medium were removed. 50 µl of complex and 25 µl DMEM containing 30% of FCS was sequentially added to the cells to obtain the final concentration of 6 nM of siRNA. After transfection, cells were incubated for ca. 30 hrs at 37 °C. *Cell lysis and RT-qPCR analysis***.** After the removal of the medium, the cells were washed with 125 µl FCW buffer / well (included in FastLane Cell Multiplex Kit, Qiagen, # 216513). Afterwards the cells were either shock frozen on dry ice and transferred to the -80 °C freezer or directly lysed. For the lysis, cells were treated for 10 minutes with the FastLane processing mix (47 µl FCPL / well + 3 ul gDNA wipeout buffer 2 / well). Carefully, 40 ul of the Lysate were transferred to a new plate to perform a heat inactivation step (6 min at 75 °C). Afterwards the lysate was diluted 1:5 in RNAse free water. One-step Duplex real-time PCR was performed using Fastlane Cell Multiplex kit (Qiagen #216513) and QuantiTect Multiplex RT-PCR kit (Qiagen #204643). Relative quantification was carried out on the ABI PRISM 7900 using Taqman gene expression assays from Applied Biosytems: hs ELAVL1_FAM _Hs00171309_m1 and an Endogenous Control of hs HPRT1_VIC _4326321E. The raw data of the Ct value were transferred into an excel template automatically by using the file load software 3.0.11. The transfection of all siRNAs were independently repeated on two assay plates. Positive

control siRNAs treatments targeting eYFP were done in triplicate on each plate. The eYFP knock down serves as a transfection control which reaches the threshold. The mean values of the two plates are presented as percent of YFP-siRNA control (serves as a kind of target-siRNA untreated control). The relative gene expression was analyzed using the delta-delta-Ct method.

Appendix Supplementary References

Buuren, B.N.M. and Wijmenga, S.S. *(***1998)** *The use of NMR methods for conformational studies of nucleic acids. Progr. NMR Spectrosc.* 32**:** 287*–387*

Case, D., Darden, T., Cheatham III, T., Simmerling, C., Wang, J., Duke, R., Luo, R., Walker, R., Zhang, W., Merz, K. and others (). *AMBER 12, University of California: San Francisco, 2012.*

Chiang, Y.-W., Borbat, P. P. and Freed, J. H. (**2005**). *The determination of pair distance distributions by pulsed ESR using Tikhonov regularization.*, J Magn Reson 172 : 279-295.

Greenfeld, M, Herschlag, D. (2013), Methods Enzymol. 2013;530:281-97. doi: 10.1016/B978-0-12-420037-1.00015-4.

Gromov, I., Shane, J., Forrer, J., Rakhmatoullin, R., Rozentzwaig, Y. and Schweiger, A. (**2001**). *A Q-band pulse EPR/ENDOR spectrometer and the implementation of advanced one- and two-dimensional pulse EPR methodology.*, J Magn Reson 149 : 196-203.

Güntert, P. (**2004**). *Automated NMR structure calculation with CYANA.*, Methods Mol Biol 278 : 353-378.

Herrmann, T., Güntert, P. and Wüthrich, K. (**2002**). *Protein NMR structure determination with automated NOE-identification in the NOESY spectra using the new software ATNOS.*, J Biomol NMR 24 : 171-189.

Hoffmann, A., Nettels, D., Clark, J., Borgia, A., Radford, S. E., Clarke, J. and Schuler, B. (**2011**). *Quantifying heterogeneity and conformational dynamics from single molecule FRET of diffusing molecules: recurrence analysis of single particles (RASP)*, Phys. Chem. Chem. Phys. 13 : 1857.

Jeschke, G., Panek, G., Godt, A., Bender, A. and Paulsen, H. (**2004**). *Data analysis procedures for pulse ELDOR measurements of broad distance distributions*, Appl. Magn. Reson. 26 : 223-244.

Jeschke, G., Chechik, V., Ionita, P., Godt, A., Zimmermann, H.; Banham, J.; Timmel, C. R.; Hilger, D. and Jung, H. (**2006**). *DeerAnalysis2006textemdasha comprehensive software package for analyzing pulsed ELDOR data*, Appl. Magn. Reson. 30 : 473-498.

Müller, B. K., Zaychikov, E., Bräuchle, C. and Lamb, D. C. (**2005**). *Pulsed interleaved excitation.*, Biophys J 89 : 3508-3522.

Pannier, M., Veit, S., Godt, A., Jeschke, G. and Spiess, H. W. (**2000**). *Dead-time free measurement of dipole-dipole interactions between electron spins.*, J Magn Reson 142 : 331-340.

Polyhach, Y., Bordignon, E., Tschaggelar, R., Gandra, S., Godt, A. and Jeschke, G. (**2012**). *High sensitivity and versatility of the DEER experiment on nitroxide radical pairs at Q-band frequencies.*, Phys Chem Chem Phys 14 : 10762-10773.

Shen, Y., Delaglio, F., Cornilescu, G., and Bax, A. (2009). *TALOS+: A hybrid method for predicting protein backbone torsion angle from NMR chemical shifts.,* J. Biomol . NMR 44 : 213-223.

Schuler, B. (**2007**). *Application of single molecule Förster resonance energy transfer to protein*

folding., Methods Mol Biol 350 : 115-138.

Tschaggelar, R., Kasumaj, B., Santangelo, M. G., Forrer, Jö., Leger, P., Dube, H., Diederich, F., Harmer, J., Schuhmann, R., García-Rubio, I. and Jeschke, G. (**2009**). *Cryogenic 35GHz pulse ENDOR probehead accommodating large sample sizes: Performance and applications.*, J Magn Reson 200 : 81-87.

Wüthrich, K. (1986) *NMR of proteins and nucleic acids.* John Wiley & Sons, New York

Zweckstetter, M. (2008) *NMR: prediction of molecular alignment from structure using the PALES software.,* Nat. Protoc. 3 : 679-690.
