# Supplementary material for: Structural basis of siRNA recognition by TRBP double‐stranded RNA binding domains
Source: EMBO J. 2018 Feb 15;37(6):e97089. doi: 10.15252/embj.201797089 (PMC5852647; doi:10.15252/embj.201797089)
Supplement: Supplementary file 2 — Expanded View Figures PDF [file EMBJ-37-e97089-s002.pdf]

Expanded View Figures

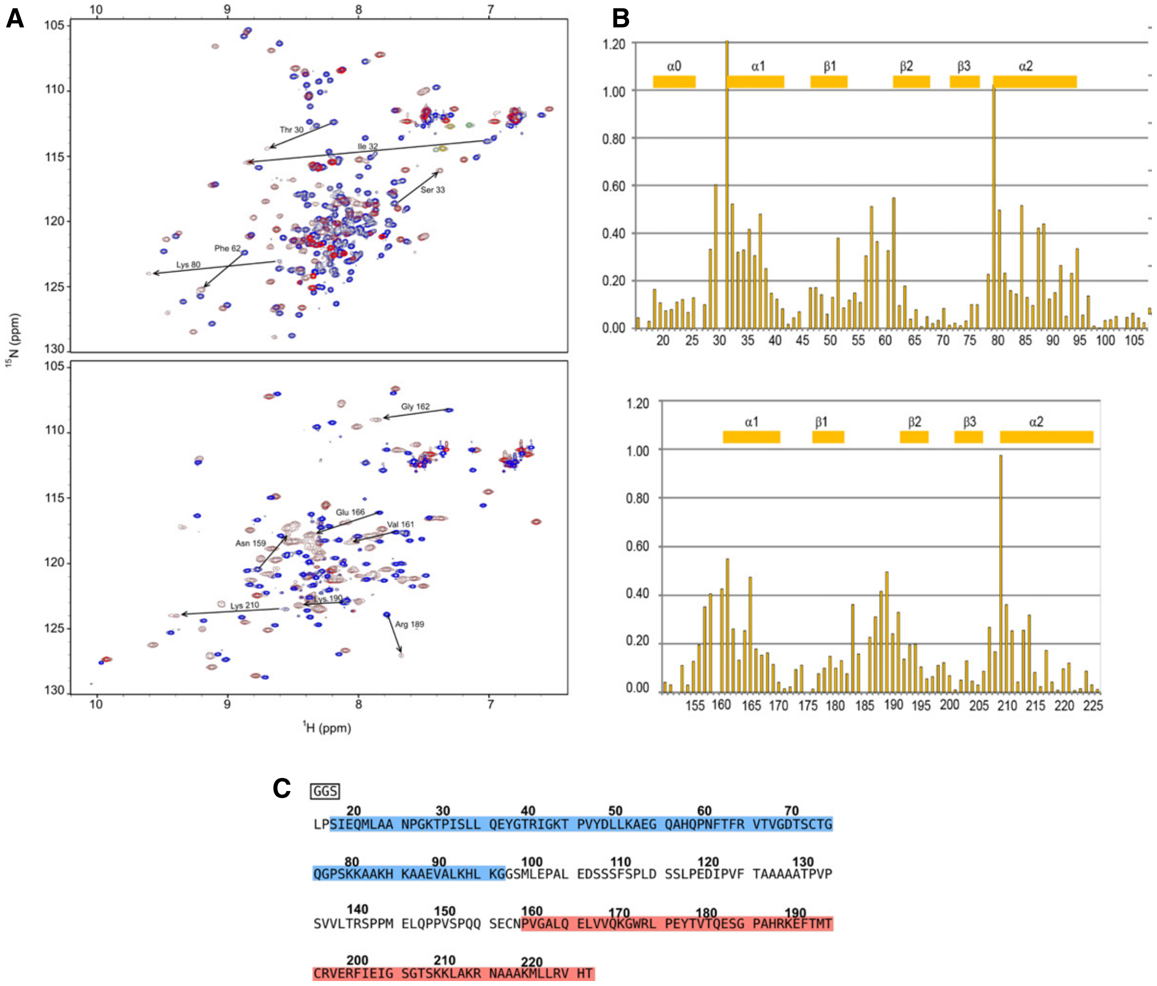

Figure EV1. Mapping of chemical shift perturbations occurring upon the formation of dsRBD1-EL86 and dsRBD2-EL86 complexes. DsRBD12 amino-acid sequence.

A ^{15}N-HSQC overlays of dsRBD-1 (upper) and dsRBD-2 (lower) in the free state (blue) and bound to dsRNA (red). Largest chemical shift perturbations observed upon EL86 binding are indicated with arrows.

B Chemical shift perturbations mapping onto the primary structures of dsRBD-1 (upper) and dsRBD-2 (lower). Gaps correspond to proline residues. Secondary structure is indicated for each domain.

C Amino acid sequence of TRBP dsRBD12. The “GGS” stretch in the black frame is coming from the expression vector. DsRBD1 (dsRBD2) regions are highlighted in blue (red).

© 2018 The Authors

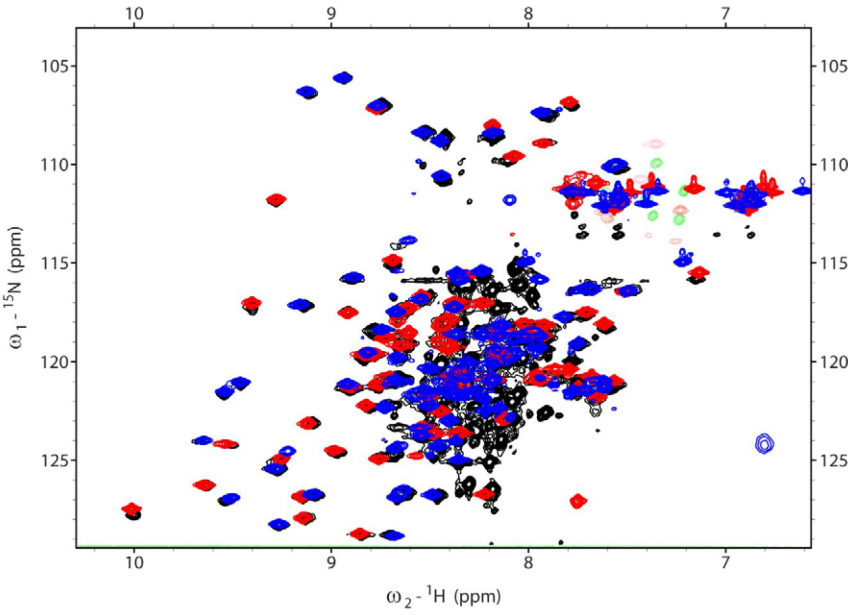

Figure EV2. Overlay of EL86-bound dsRBD1, dsRBD2, and dsRBD12 fingerprints.

The ^{15}N-HSQC spectra of dsRBD1, dsRBD2, and dsRBD12, are shown in blue, red, and black, respectively. All three spectra were recorded in the presence of dsRNA (EL86). Unmatched black peaks correspond to the residue linker between dsRBD1 and dsRBD2.

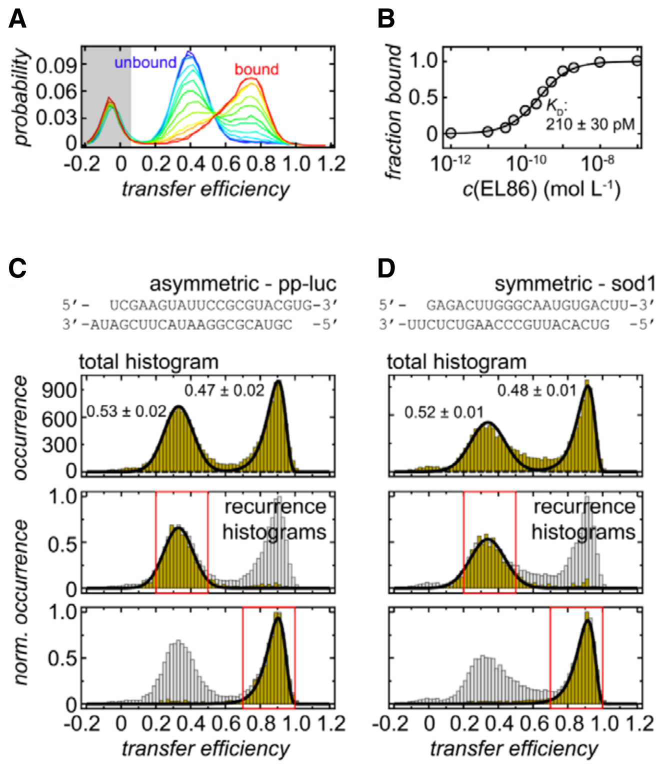

Figure EV3. TRBP-RNA binding isotherm and sequence dependence of RNA binding as characterized by single-molecule FRET.

A Normalized transfer efficiency histograms of Cy3B-CF660R-labeled dsRBD12 in the presence of different EL86 RNA concentrations. Free dsRBD12 (blue) can be distinguished from RNA-bound dsRBD12 (red) via its transfer efficiency. The peak in the gray-shaded area originates from molecules without an active acceptor fluorophore and is not included in the analysis.

B RNA binding curve generated from the plots shown in panel (A). The experimental data were fitted to a binding isotherm (solid line).

C, D Transfer efficiency histograms of CF660R-labeled dsRBD12_22-235 C158S in complex with Cy3B-labeled pp-luc and sod1. Top: Transfer efficiency histograms reveal two subpopulations that are equally populated. Errors associated with their estimates from multiple measurements. Bottom: Recurrence transfer efficiency histograms (Hoffmann *et al*, 2011) were used to extract subpopulation-specific fit parameters for the peak shapes. Red boxes highlight the initial transfer efficiency range used for the analysis. The recurrence interval *T* was set to (0, 10 ms). See Materials and Methods section for details.

© 2018 The Authors

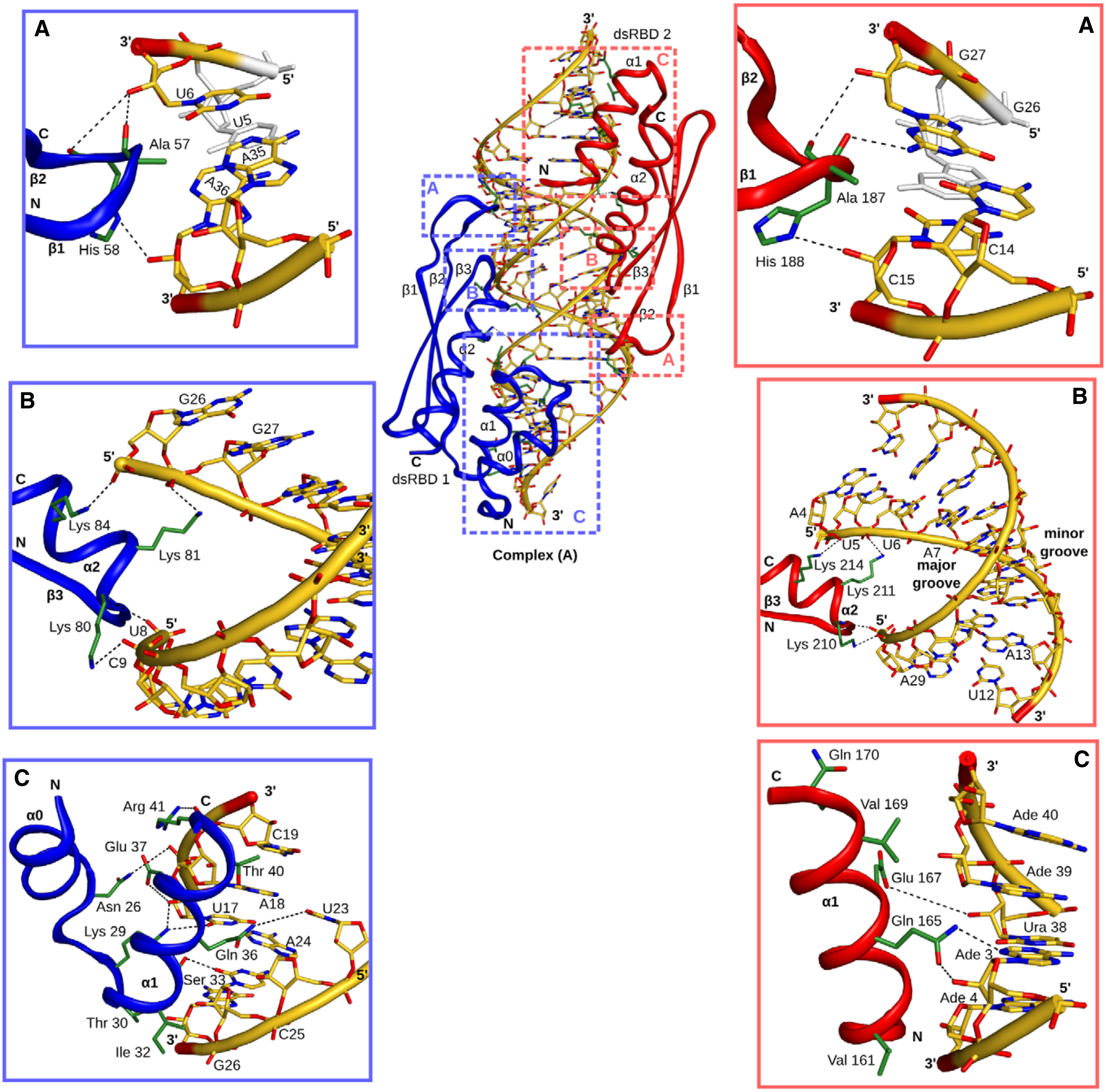

Figure EV4. Interactions between dsRBD12 and EL86.

A–C A representative structure of complex A is shown with dsRBD1 and dsRBD2 colored in blue and red, respectively. Protein side-chains and RNA are represented as green and yellow sticks, respectively. Polar interactions are displayed with dashes. The interactions between EL86 and the β1–β2 loop, KKxAK motif, and helix α1 of dsRBD1 (blue) and dsRBD2 (red), are shown in details in panels (A–C), respectively.

© 2018 The Authors

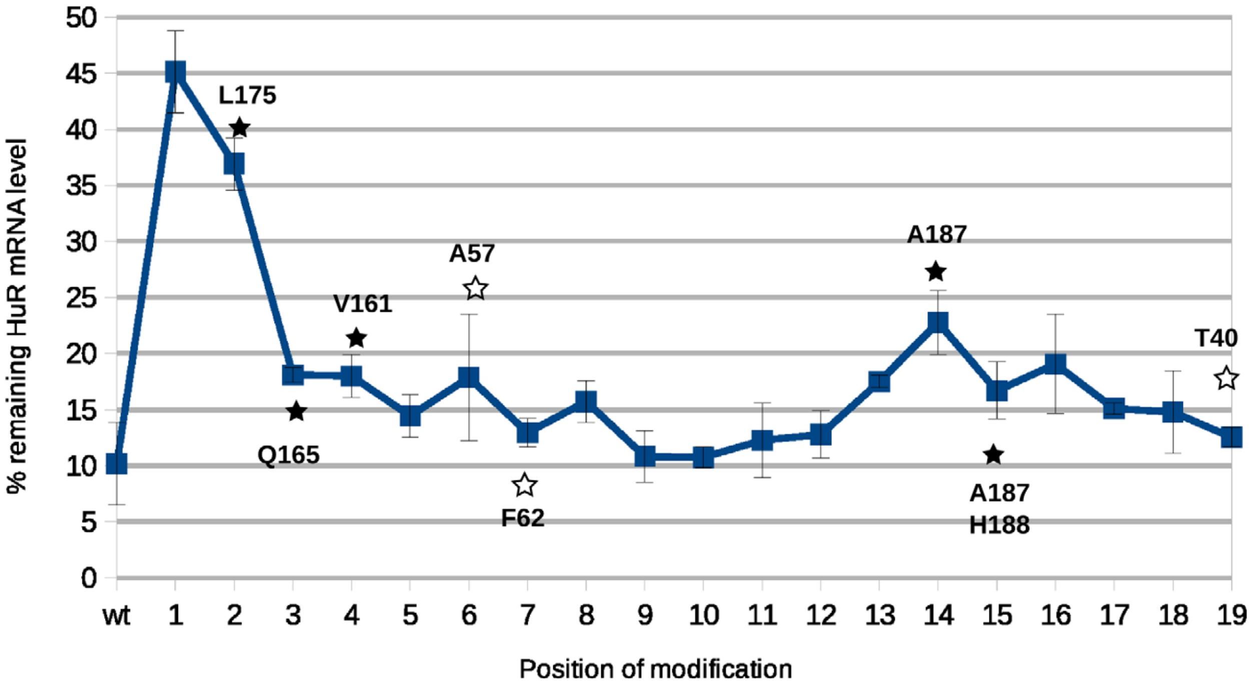

Figure EV5. Impact of EL86 (guide strand) 2′-MOE modifications on the knockdown efficiency of endogenous ELAVL1 mRNA in HeLa cells.

Remaining mRNA level (*y*-axis) was quantified by RT–qPCR (mean ± SD from two replicates). The positions of the modified residues are shown on the *x*-axis. "wt" is the mRNA level quantified for unmodified EL86. Contacts between dsRBD12 and EL86 observed in our NMR structure (complex A) are shown with white (black) stars for those involving dsRBD1 (dsRBD2).

 © 2018 The Authors
